# Parenting stress after perineal tear during childbirth: the role of physical health and depressive symptoms

**DOI:** 10.3389/fpsyg.2025.1477316

**Published:** 2025-04-15

**Authors:** Valentine Rattaz, Sarah Cairo Notari, Valérie Avignon, Chahin Achtari, Antje Horsch

**Affiliations:** ^1^Institute of Higher Education and Research in Healthcare, University of Lausanne, Lausanne, Switzerland; ^2^Faculty of Psychology and Educational Sciences, University of Geneva, Geneva, Switzerland; ^3^Department Woman-Mother-Child, Lausanne University Hospital and University of Lausanne, Lausanne, Switzerland

**Keywords:** parenting stress, perineal tear, physical health, depression, childbirth

## Abstract

**Introduction:**

This study investigates the impact of the severity of perineal tear during childbirth on parenting stress at 10–12 weeks postpartum. Studies have shown that up to 80% of primiparous mothers may suffer a perineal tear, which can have important physical and psychological consequences. As the adjustment to parenthood can be highly demanding and stressful for primiparous parents, we hypothesized that having experienced a perineal tear during childbirth could be an additional stressor reducing the resources to cope, which could increase parenting stress. We also hypothesized that this increase in parenting stress could be explained by the consequences of the perineal tear on mothers’ physical health and depressive symptoms.

**Methods:**

This study included 155 primiparous mothers with various degrees of perineal tear (from an intact perineum to a 4th degree tear).

**Results:**

Results showed that perineal tear was not directly associated with parenting stress at 10–12 weeks postpartum. However, we found an indirect pathway of influence through mothers’ physical health and depressive symptoms. Higher degrees of perineal tear were associated with worse physical health in mothers causing an increase in depressive symptoms that, in turn, was associated with higher parenting stress.

**Discussion:**

The results indicate that perineal tear is indirectly associated with parenting stress, through its influence on physical health and depressive symptoms. Therefore, it seems crucial to focus on managing the physical consequences of perineal tear in the first weeks postpartum, to avoid detrimental consequences on mothers’ mental health and parenting.

## 1 Introduction

Becoming a parent is a stressful and challenging life transition for first time parents (Cowan et al., 1991; Kuersten-Hogan and McHale, 2021). It is a period marked by hormonal changes (Saxbe et al., 2018), recovery from the childbirth (Baghirzada et al., 2018), changes in daily routines (Lévesque et al., 2020), sleep deprivation (Medina et al., 2009), shifts in social roles (Haynes, 2008), and changes in the couple relationship (Doss and Rhoades, 2017). Parents also need to learn how to understand their infant’s signals and needs in order to develop their parental competencies and a positive parent-infant relationship (Javadifar et al., 2016; Deave et al., 2008). Parents need resources to cope with this physically and emotionally demanding period. Internal resources, such as a good self-esteem, a good sense of competence, or adaptative coping strategies, and external resources, such as social support from family and friends, and support from healthcare providers, have been identified as key elements to help parents in this transition (Ben-Ari et al., 2009; Barimani et al., 2017). If the resources available to the parent are not sufficient to cope with the parenting demand, parenting stress can arise (Deater-Deckard and Scarr, 1996). Parenting stress is defined as the “aversive psychological reaction to the demands of being a parent” (Deater-Deckard, 1998), p. 314. The negative feelings are directly attributable to the demand of parenthood, which makes it distinct from general stress. High levels of parenting stress can negatively affect the way parents interact with their infant. For example, it has been shown that a higher level of parenting stress is associated with lower maternal sensitivity (i.e., the ability to perceive and respond adequately to the child’s signals) during infancy (Booth et al., 2018; Dau et al., 2019). Moreover, parenting stress in the first years after birth can have detrimental consequences on the child. Indeed, parenting stress during infancy has been shown to be associated with more emotional and behavioral problems at 3 years of age (Hattangadi et al., 2020). Studies have shown that parenting stress at 3 months postpartum is positively associated with parenting stress at 7 and 14 months postpartum (Thomason et al., 2014) and tends to increase during the first years after birth (Barboza-Salerno, 2020). These results suggest that parents who feel stressed in the first weeks postpartum might therefore be at risk of remaining, or becoming even more, stressed over time. This underlines the importance of identifying the factors in the early postpartum period that can contribute to parenting stress in order to act on these factors and reduce the stress.

Perineal tears constitute a physical and a psychological stressor that occur in the already highly stressful period of life that is the transition to parenthood. Perineal tear is an injury to the tissues around the vagina and anus that can happen during childbirth. It is a common childbirth complication, affecting 70 to 80% of primiparous mothers and 50 to 60% of multiparous mothers (Samuelsson et al., 2002; Jansson et al., 2020; de Castro Monteiro et al., 2016). Perineal tears are classified according to the severity of the injury, from mild (1st or 2nd degree tear) to severe (3rd or 4th degree tear). First-degree tear involves a tear of the skin and the tissues directly beneath the skin of the perineum and the vaginal mucosa; second-degree tear, which is the most common type, involves the skin and the muscle of the perineum and the vaginal mucosa; third-degree tear involves an injury from the perineum to the anal sphincter without damage to the anal mucosa; finally, fourth-degree tear, which is the least common type, involves a damage from the perineum to the anal sphincter and into the rectum and the anal mucosa (Goh et al., 2018). These injuries can have consequences on mothers’ physical functioning, with long lasting damages to the pelvic floor muscles, perineal pain, incontinence, and dyspareunia (Gommesen et al., 2019a; Huber et al., 2021; Gyhagen et al., 2015). If around 80% of mothers will physically recover from their perineal tear in the 12-months after birth (Bagade and Mackenzie, 2010; No, 2015), some studies have shown that some mothers will continue experiencing physical consequences of perineal tears up to 10 years (Pollack et al., 2004; Viannay et al., 2021). This suggests that there could be some lifelong physical consequences of perineal tear. Perineal tears are also psychologically highly distressing (Beck, 2021; O’Reilly et al., 2009). Mothers who experienced perineal tear are reporting feelings of shame, isolation, sadness, or depression (Lindberg et al., 2020; Priddis et al., 2013), and are more likely to develop postpartum depressive symptoms compared to mothers with no perineal tear (Lestari and Istiqomah, 2022). The extent of these physical and psychological consequences is related to the severity of the perineal tear (Huber et al., 2021), the more severe the tear, the more negative the consequences. Although the physical and psychological consequences of a perineal tear have been widely studied, what is still unknown is the impact of these consequences on parenthood, and in particular, on parenting stress.

Among the factors influencing parenting stress, studies have shown that mothers’ good physical health is related to less parenting stress (Matvienko-Sikar et al., 2018). Parenting in the early postpartum is physically exhausting (Martins, 2019), as mothers are sleep-deprived and still recovering from the physical consequences of childbirth. Indeed, the baby needs to be fed at regular intervals during the day and night, and often needs to be carried and rocked, which requires physical resources. Childbirth is often associated with a decrease in mothers’ perceived health (Song et al., 2014), especially after severe perineal tear (Priddis et al., 2013). Mothers who experienced perineal injuries, even minor, report having persistent perineal pain 3 months after childbirth, with a prevalence that can go from 17% for minor injuries to 75% for severe perineal injuries (Åhlund et al., 2019). Persistent perineal pain has been showed to interfere with mothers’ daily activities, including childcare activities (Bijl et al., 2016; Molyneux et al., 2024). These physical limitations associated with the pain can cause a decrease in maternal sense of competence (Srisopa and Lucas, 2021) and increase parenting stress. Psychological health is also associated with parenting stress. Parental psychological health is a resource that facilitates coping with life events, such as the transition to parenthood (Perren et al., 2005). Studies have shown that higher postpartum depressive symptoms were associated with higher parenting stress (Reck et al., 2016; Leigh and Milgrom, 2008; Kerstis et al., 2016). Depressive mothers perceived their infant as more difficult (Sechi et al., 2020; Slomian et al., 2019) and perceived themselves as less competent in infant care (Dlamini et al., 2023; Slomian et al., 2019). Of course, physical and psychological health are not independent from one another but rather influence each other. Indeed, mothers reporting perineal pain in the weeks following childbirth are more at risk for postpartum depression (Gaudet et al., 2013; Chang et al., 2016; Lim et al., 2020).

Surprisingly, to date, no study has investigated the impact of a perineal tear on parenting stress in the weeks following childbirth, even though perineal tears are one of the most common childbirth complications, constitute a major stressor for mothers, and have important negative consequences on their physical (Gommesen et al., 2019a; Huber et al., 2021; Gyhagen et al., 2015), mental (Dunn et al., 2015; Lestari and Istiqomah, 2022; Baumann et al., 2023), or sexual (Gommesen et al., 2019a; Sayed Ahmed et al., 2017; Rathfisch et al., 2010) health. All of these aspects have been considered in various fields of study (e.g., medicine, psychiatry, sexual health) but have not been investigated from the perspective of family functioning, i.e., the effect that having suffered a perineal tear may have on the mother’s experience of parenting. It is conceivable that, in the challenging period that is the transition to parenthood, the stress induced by the perineal tear, along with its negative physical and psychological consequences, might reduce the resources that women have to cope with the demand of parenting, which put them at risk for experiencing parenting stress. The aim of the present study was therefore to investigate the association between the degree of perineal tear and parenting stress in mothers at 10 to 12 weeks postpartum, and to investigate the mediation effect of both physical health and depressive symptoms in this association. We hypothesized that having suffered a perineal tear will be associated with parenting stress, such that a higher degree of tear will be predictive of higher parenting stress. As perineal tears have an influence on physical health and depressive symptoms, we hypothesized that they could both be mediators of this association.

## 2 Methods

### 2.1 Sample

The present study included 155 primiparous mothers who gave birth at the Lausanne University Hospitals and is part of a larger study on the impact of perineal tear on childbirth-related posttraumatic stress disorder (CB-PTSD) and depression. The sample size calculation was determined based on the expected differences in CB-PTSD and depression between the clinical group of women with a perineal tear and a control group of women without a perineal tear, assuming a confidence level of 95% and a power of 80%, leading to required sample size between 138 and 179 mothers. To be included in the study, mothers had to be 18 years old or older, had to have given birth to a unique healthy term baby (≥ 37 weeks of gestational age) by vaginal delivery and speak French sufficiently enough to complete questionnaires in French.

### 2.2 Procedure

Primiparous mothers were recruited during their hospital stay after childbirth (3–5 days) by a midwife in the maternity ward or during a specialist consultation for women who had a severe perineal tear. The study was explained to the mothers and, if they agreed to participate, they were given an information sheet and consent form regarding the study to sign. At 10–12 weeks postpartum, mothers received a set of self-report questionnaires at home with a stamped envelope to be returned to the Lausanne University Hospitals. The data from the questionnaires were then entered into the electronic data capture tool REDCap (Harris et al., 2019; Harris et al., 2009) and the questionnaires were stored in a locked cabinet accessible only by the research investigators. Maternal and infant medical data was extracted from the hospital records. Sociodemographic information and data regarding maternal weight and height were reported by the mothers at 10–12 weeks postpartum. This study received the approval of the local ethics committee (2017-01565).

### 2.3 Measures

#### 2.3.1 Degree of perineal tear

The degree of perineal tear, from an intact perineum to 4th degree-tear, was obtained from the birth record in the mothers’ medical file.

#### 2.3.2 Parenting stress

Parenting stress was measured using the French version of the Parenting Stress Index Short-Form (PSI-SF; Toucheque et al., 2016). The PSI-SF is a 36-item self-report questionnaire assessing three domains of parenting stress: parental distress, which assesses the emotional distress felt in parenting; parent–child dysfunctional interaction, which assesses the parent’s perception of the relationship; and difficult child, which assesses the parent’s perception of the child as being behaviorally difficult. The total stress score is obtained by summing the score of the three subscales. A cut-off value of 80–89 may be used to determine highly stressed parents and a score above 90 may be considered as clinically significant (Abidin, 1995). The present study used the total stress score as an assessment of the general stress perceived by the mother regarding parenting, a higher score on the PSI-SF being indicative of high parenting stress.

#### 2.3.3 Postpartum depression

Depression was assessed using the French version of the Edinburgh Postnatal Depression Scale (EPDS; Guedeney and Fermanian, 1998). The EPDS is a 10-item self-report questionnaire to assess depressive symptoms in the last 7 days. The total score is obtained by summing the score of each item. A cut-off value of 11 or higher is recommended to identify respondents who might meet diagnosis criteria for postpartum depression (Levis et al., 2020). For the present study, the total score was used, a higher score on the EPDS being indicative of more depressive symptoms.

#### 2.3.4 Maternal physical health

Physical health was assessed using the Short Form 36 Health Survey (SF-36; Perneger et al., 1995). The SF-36 is a 36-item general health instrument assessing both physical health and mental well-being; only the physical health score was used in the present study. The summary measure of physical component of the SF-36 is assessed by measuring four domains of physical health: physical functioning, role limitation due to physical problems, bodily pain, and general health perception. The physical health score is obtained in three steps: first, each item is recoded in a 0–100 scale; second, a mean score is obtained for each of the four domains; third, the mean of the four domain is calculated to obtain the physical health score. A cut-off value of 60 or higher has been suggested to conclude to a good physical health (Bieleman et al., 2009). For the present study, the total score SF-36 was used, a higher score being indicative of a better physical health.

## 3 Statistical analyses

Descriptive statistics and correlational analyses were obtained using IBM SPSS Statistics 29.0 and structural equation modeling tested the mediation models using Mplus 8.3 (Muthén and Muthén, 2017).

We tested an overall multiple mediator model with both parallel and serial pathways to explore how the two mediators might work together. Parallel mediation assumed that both physical health and depressive symptoms are mediators of the relationship between the degree of perineal tear and parenting stress, whereas serial mediation assumed a causal association between the mediators, with a specified direction (Hayes, 2013). In this case, the serial mediation assumed that the influence of physical health on depressive symptoms had a mediation effect on the relationship between the degree of perineal tear and parenting stress.

The significance of indirect effects was estimated with a maximum likelihood estimator and using the bias-corrected bootstrap test procedure (MacKinnon et al., 2004). The data were resampled 5,000 times, enabling the mediating effect to be calculated at a 95% confidence interval (CI). Statistical significance was achieved when lower bound (LL) and upper bound (UP) CI did not include zero. As the tested model was a fully saturated path model, no model fit indices were reported, as a saturated model has a perfect fit.

Of note, the present analysis did not follow the causal steps approach of mediation analysis (Baron and Kenny, 1986), which postulates that a mediation analysis can only be tested if there is a significant association between the independent variable (X) and the dependent variable (Y). Indeed, even if X and Y are not associated, a mediator (M) can still have an indirect effect from X to Y through M (Hayes, 2009; Zhao et al., 2010). Therefore, the path between X and Y should not be a prerequisite for further examination of the indirect paths. If there is no association between X and Y, we will refer indirect effects rather than referring to a mediation effect.

## 4 Results

Descriptive statistics of the study variables can be found in Table 1. The mean age of the women was 31.54 years old (SD = 3.69). Most of them were married (68.4%) and were highly educated, with 62.6% of them having completed a university degree. Regarding perineal tear, 58 mothers had an intact perineum (37.4%), 28 had a first-degree tear (18.1%), 36 had a second-degree tear (23.2%), 31 had a third-degree tear (20%) and 2 had a fourth-degree tear (1.3%). Regarding parenting stress, 7.6% of mothers were considered as highly stressed (score ranged between 81 and 89) and 4.2% were considered as clinically stressed (score above 90). Regarding postpartum depression, 22% of mothers had a score above the cut-off 11. Finally, regarding physical health, 23.9% of mothers were considered as having a low physical health (score under 60), and the mean value was below normative values from Switzerland (Roser et al., 2019).

**Table 1:** Descriptive statistics of the study variables.

	*N*	Min.	Max.	Mean	SD
Mothers’ age	155	22	43	31.54	3.69
Mothers’ weight (kg)	152	43	112	66.95	12.67
Mothers’ height (cm)	155	150	189	166.45	6.46
Newborn’s weight (g)	155	2,530	4,280	3325.81	366.75
Newborn’s height (cm)	155	43	54	49.79	2.03
Parenting stress	155	40	114	61.64	15.39
Physical health	155	35.63	97.50	72.73	15.71
Depressive symptoms	155	0	21	6.74	4.99

As our analyses relied on self-report data, we conducted Harman’s single factor test to assess the common method bias. Results of the test showed that the first factor accounted for 18.79% of the total variance, below the 50% threshold, indicating no common method bias.

Spearman correlations, suitable for non-normally distributed data such as in our sample, were conducted between the variables of interest and can be found in Table 2. As there were significant positive correlation between the variables of interest and the mother’s age, we included this variable in the mediation model. We also included the baby’s weight, as a high birth weight is known to be a risk factor of perineal tear (Gommesen et al., 2019b).

**Table 2:** Spearman correlation matrix for study variables.

	1	2	3	4	5	6	7	8	9
Mother’s height	1								
Mother’s weight	0.332^**^	1							
Mother’s age	0.016	−0.048	1						
Newborn’s size	0.036	0.214^**^	0.008	1					
Newborn’s weight	0.236^**^	0.305^**^	0.013	0.625^**^	1				
Degree of tear	−0.005	0.066	0.108^*^	0.062	0.025	1			
Parenting stress	0.087	0.071	0.004	−0.041	0.059	0.016	1		
Depressive symptoms	0.002	0.125	−0.020	−0.023	−0.021	0.102	0.623^**^	1	
Physical health	0.026	−0.110	0.019	−0.094	−0.192^*^	−0.170^*^	−0.287^**^	−0.353^**^	1

^*^p < 0.05 and ^**^p < 0.01.

**Figure 1:**
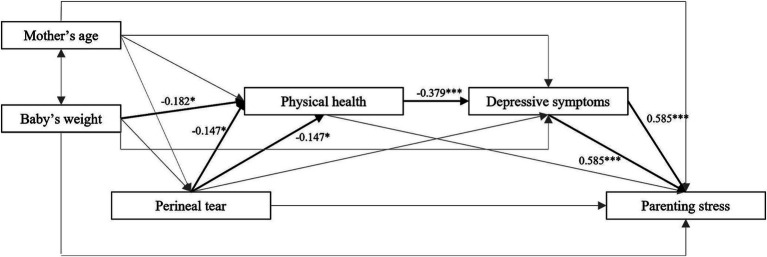
Mediation model including standardized estimates. Bold arrows represent significant associations. ^*^p < 0.05, ^**^p < 0.01, and ^***^p < 0.001.

The estimated models and the significant associations between the variables can be found in Figure 1. Results showed no significant direct effect of perineal tear on parenting stress. The newborn’s weight had a significant effect on maternal physical health, with a higher newborn’s weight being associated with lower maternal physical health. Concerning the links between perineal tear and the mediators, results showed an effect of perineal tear on maternal physical health, with a higher degree of perineal tear predicting lower maternal physical health, but not on maternal depressive symptoms. Results also showed an effect of maternal physical health on maternal depressive symptoms, with lower physical health being associated with higher depressive symptoms. Finally, maternal depressive symptoms influenced parenting stress, with higher depressive symptoms being associated with higher parenting stress. Regarding the significance of the three indirect effects of interest, results showed that the indirect pathway from perineal tear to parenting stress through physical health was not significant (*b* = 0.150, 95%CI[-0.085, 0.633]). The indirect pathway through depressive symptoms was not significant (*b* = 0.048, 95% CI [−1.033, 1.154]). Finally, results showed that the indirect serial pathway from perineal tear to parenting stress through the influence of physical health on depressive symptoms was significant (*b* = 0.418, 95% CI [0.033, 0.991]). As the direct effect of perineal tear on parenting stress was not significant, the results suggest that there is only an indirect effect through the impact of maternal physical health on maternal depressive symptoms when investigating the association between perineal tears and parenting stress.

## 5 Discussion

The aim of the present study was to investigate the association between the degree of perineal tear and parenting stress, as well as the indirect effects of maternal physical health and depressive symptoms. We found no direct association between the degree of perineal tear and parenting stress. However, results showed that perineal tear could influence parenting stress through its effect on physical health and, in turn, on depressive symptoms.

We hypothesized that perineal tear could constitute an additional significant stressor for mothers, increasing the stress load of primiparous mothers in the first weeks postpartum, which would result in an increase in parenting stress. Our results did not confirm this hypothesis. The absence of a direct association between perineal tear and parenting stress could be explained by the perception that mothers may have of their perineal tear. Indeed, it is possible that not all mothers experienced their perineal tear as a significant source of stress. Previous studies have shown that the stress felt by mothers following childbirth is related to the evaluation of their childbirth experience as more or less positive (Hinic, 2017; Holopainen et al., 2020; Molgora et al., 2020). In the context of the present study, mothers may have suffered a perineal tear but still have experienced childbirth positively and be satisfied with the medical management of their injury, which would not increase the stress load. Therefore, more than the degree of the tear itself, it could be mothers’ perception and the way they experienced the tear, as more or less stressful, that could have an influence on parenting stress. Further studies need to investigate mothers’ perception of their injury and their childbirth experience in relation to parenting stress in the first weeks postpartum. This would also mean that if the stress is related to the perception of the perineal tear, there is room to reduce the stress. Studies have shown that perineal tear and its consequences is poorly understood by mothers (Crookall et al., 2018) and that support and advice from healthcare providers could reduce the distress felt by mothers after a severe perineal tear (Williams et al., 2005). This underlines an important implication for the clinical practice, as discussing with mothers about their experience, answering the questions they might have, and enhancing their knowledge about the injury could reduce their stress. Moreover, as mentioned in the introduction, the perceived support from healthcare providers has been identified as an important resource for mothers in the transition to parenthood that could reduce parenting stress. Therefore, providing informational and emotional support to mothers seems highly important. This also implies that the support to be provided must be multidisciplinary, and that healthcare professionals must be made aware of these issues and trained to offer this support.

We also hypothesized that physical health and depressive symptoms would be indirect paths through which the degree of perineal tear could influence parenting stress. Results suggest that the degree of perineal tear influences mothers’ physical health, highlighting the physical consequences of the severity of perineal tear in the following weeks after childbirth. In terms of clinical implications, this suggests that we need to ensure that mothers have access to long-term follow-up after a perineal tear in to identify the potential implications for their physical health, which includes any physical limitations they could have, any persistent pain or perception of a poorer physical health. This is of particular importance because, even though the present study did not confirm the previous findings on the role of physical health on parenting stress, results showed that mothers’ poorer physical health was associated with higher depressive symptoms. Indeed, depressive symptoms were not associated with the degree of perineal tear, but only with women’s physical health. The postpartum period is a time when mothers can easily struggle with their mental health (O’Hara and Wisner, 2014) and the perception of a poorer health could increase these difficulties. The feeling of not having fully recovered from childbirth may add an extra layer of worry in an already overwhelming period. According to our results, this increase in depressive symptoms can, in turn, influence parenting stress. This indirect path between the severity of the perineal tear on parenting stress through the impact of physical health on depressive symptoms underlines the complexity of the factors that could play a role in parenting stress in the postpartum period. There is a need to consider the physical and psychological risk factors that can accumulate in the postpartum period after enduring a perineal tear.

Regarding clinical implications, it is important to note that perineal tears are frequent in vaginal delivery. However, the present study draws our attention to the fact that it can indirectly impact parenting stress, which might negatively affect the relationship with the infant, and, consequently, the child’s development. Indeed, parenting stress can undermine parents’ ability to be sensitive to the child’s needs (Dau et al., 2019) and can increase the use of harsh parenting behavior (Jackson and Choi, 2018). Moreover, as parenting stress tends to increase in the first years after birth (Barboza-Salerno, 2020), chronic parenting stress can later turn into parental burnout (Mikolajczak and Roskam, 2018), which can lead to an emotional detachment from the child (Mikolajczak and Roskam, 2020). These adverse consequences underline the importance of building an understanding of the multiple factors involved in parenting stress, to which this study contributes. The results also provide an indication of the factors on which it is possible to intervene to reduce this indirect effect. As mentioned, perineal tears occur frequently; thus, there is a need to focus on the best possible healing to reduce the potential impact of the tear on physical health. Although there are numerous care protocols for the management of perineal tear that have provided some level of evidence on the days after birth (Gondim et al., 2025; Kurnaz et al., 2025; White and Atchan, 2022), there is no evidence for the efficacy of these interventions beyond this acute period (White and Atchan, 2022). Further studies should be conducted to identify interventions that can help mothers in the long term and prevent the negative consequences of poor perineal repair.

The present study has some limitations. First, the small sample included in the study, and the fact that it was recruited from a single hospital, and therefore from a single region of Switzerland, could limit the generalizability of the results. However, the fact of having recruited from a single hospital ensures a degree of standardization in the protocol for managing perineal tears, since the women are all monitored in the same place. Second, we had no medical data on the repair of the perineal wound. Even though specific protocols exist to standardize the repair of perineal tears, some injuries might have healed better than others and this could have had an influence on the longer-term consequences of the tear. We also lack information about pain management mothers received or used. Third, although the SF-36 is an indicator of general physical heath, we lack a measure of perineal pain or pain specifically related to childbirth. Finally, we had no measurement of prenatal maternal stress or physical health. As we conceptualized our hypothesis based on an aspect of cumulative stress, having information about the stress that might already be felt by the mother, or the tendency of being easily stressed, as well as her physical health before childbirth could have added helpful information. This limitation encourages further studies to investigate this cumulative effect of stressors in a prospective study design. Further studies should also look consider the role of the partner, as the emotional and instrumental support they provide could be a protective factor for the mothers. Despite these limitations, this study is the first to investigate the association between perineal tear and parenting stress in the first weeks after childbirth. This innovative aspect needs to be highlighted, as it provides further information on the childbirth-related factors that can influence parenting stress.

## 6 Conclusion

To date, this is the first study investigating the association between perineal tear and parenting stress. The results suggest that healthcare providers should be particularly attentive to the physical consequences of perineal tears, as it seems to be the starting point of a cascade effect that can increase depressive symptoms and, in turn, parenting stress. Reducing the physical impact of perineal tear by making sure that women can recover as well as possible could help defuse this cumulative effect to decrease parenting stress and its negative consequences on the parent and the child.

## Data Availability

The raw data supporting the conclusions of this article will be made available by the authors, without undue reservation.
